# Quality Control of *Psoralea corylifolia* L. Based on High-Speed Countercurrent Chromatographic Fingerprinting

**DOI:** 10.3390/molecules25020279

**Published:** 2020-01-09

**Authors:** Xiaoxue Wu, Xuemin Gao, Xinmei Liu, Shuyi Zhang, Huayu Yang, Xuan Zhu, Hua Song, Funan Li, Qing Chen

**Affiliations:** Fujian Provincial Key Laboratory of Innovative Drug Target Research, School of Pharmaceutical Sciences, Xiamen University, Xiamen 361002, China; xiaoxue_wu1@126.com (X.W.); holygxm@xmu.edu.cn (X.G.); liuxinmei6427@163.com (X.L.); shuyi_zhang95@163.com (S.Z.); huayu_yang@126.com (H.Y.); zhuxuan@xmu.edu.cn (X.Z.); songhua@xmu.edu.cn (H.S.); fnlee5@xmu.edu.cn (F.L.)

**Keywords:** HSCCC, fingerprint, *Psoralea corylifolia* L., quality control

## Abstract

Traditional Chinese medicine (TCM)has played an important role in promoting the health of Chinese people. The TCM *Psoralea corylifolia* L. has been used in the treatment of various kinds of diseases including enuresis, vitiligo, and calvities. However, therapeutic effects of *P. corylifolia* L. have often influenced by the quality of plants. So, it is very important to control the quality of *P. corylifolia* L. In this study, analytical high-speed countercurrent chromatography (HSCCC) was successfully used to fingerprint *P. corylifolia* L. Samples of *P. corylifolia* L. were extracted by ultrasonic extraction. *n*-hexane-ethyl acetate–methanol–water at a ratio of 5:5.5:6.5:5 (*v*/*v*) was selected as a two-phase solvent system and the condition of HSCCC were optimized in order to good separation. And the method of HSCCC was verified (reproducibility, precision, and stability). HSCCC chromatograms exhibited six common peaks, which were selected as indicator compounds for the quality control of *P. corylifolia* L. Within 20 types of medicinal materials, chemical components are similar, but the levels of components are quite different in HSCCC fingerprint. The present results demonstrate that the HSCCC method provides a reliable basis for the quality control of *P. corylifolia* L. and can also be applied to confirm the authenticity of plant materials.

## 1. Introduction

**Figure 1:**
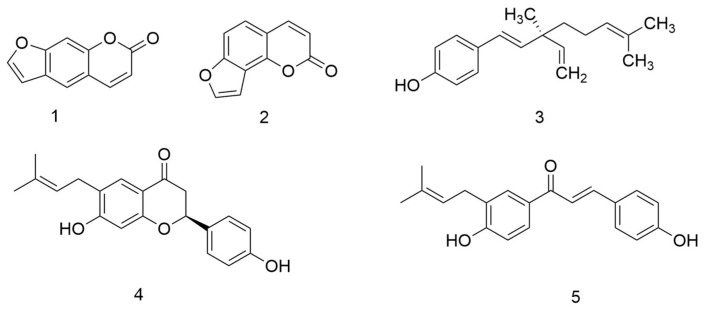
The structure of effective components of *Psoralea corylifolia* L. Compounds depicted above include (**1**) psoralen, (**2**) isopsoralen, (**3**) bakuchiol, (**4**) bavachin, and (**5**) isobavachalcone.

The dry fruits of leguminous plant *Psoralea corylifolia* L. (Buguzhi in Chinese) are one of the most popularly used plant materials in TCM. *P. corylifolia* L. has been used for the treatment of various diseases including enuresis, vitiligo, pollakiuria, asthma, cough, nephritis, and calvities [1]. It exhibits many biological activities including anticancer [2], anti-aging [3], anti-osteoporosis [4], and other activities [5,6]. The active components of *P. corylifolia* L. are coumarins [7], such as psoralen and isopsoralen (Figure 1). Pharmacological tests have revealed that psoralen and isopsoralen have antioxidant [8], antitumor [2], and antibacterial [9] activities. Flavonoids contained within *P. corylifolia* L. also have biological activities. Flavonoid extracts of *P. corylifolia* L. exhibited osteoblastic proliferation-stimulating activity on UMR106 cells [10].

However, differences in the geographic origin, climate, and local environmental conditions of plants significantly affect the chemical composition of *P. corylifolia* L., which leads to different effects. Further, since selling the highly demanded plant is profitable, the TCM market is saturated with products that contain mixtures of spurious and genuine plant materials. Low-quality materials are often falsely represented as high-grade ones in an effort to inflate profits. Counterfeits of *P. corylifolia* L. discovered on the market have included *Abutilon theophrasti* Medic. and *Crotalaria pallida* Ait. These plants are similar to *P. corylifolia* L. in terms of their morphological characteristics but they can be distinguished by their chemical constitutions. Quality problems influence the treatment of TCM. The determination of a single or even several components within a plant does not go far enough to evaluate the chemical composition of plant material. These problems require a method that can be used to differentiate between TCM and counterfeits through the analysis of their chemical components. Fingerprint analysis can be used as a method to evaluate the holistic quality of plants used in TCM. The method can be used to verify the authenticity of medicinal products and can also be used to evaluate the quality of TCM.

Traditional Chinese medicine contains a large number of herbal medicinal and Chinese patent medicinal products, each of which contains many compounds that may be relevant to the biological activity of the remedy. Because of complex sources of medicinal products, quality control in TCM has become an urgent issue. Recently, a fingerprint technique has been suggested as a meaningful method for controlling quality of TCM. And it has been increasingly attracting the attention of researchers [11]. It is widely used in TCM for quality control. The fingerprint technique emphasizes the systemic characterization of the composition of samples and has been used to identify and assess the stability of TCM [12]. The TCM fingerprint is a chromatogram or spectrogram that defines the holistic chemical characteristics of TCM by analytical means after proper processing. Currently, fingerprint analysis has been introduced and accepted by the WHO as a strategy that can be used for the assessment of herbal medicines [13]. And it is also required by the Drug Administration Bureau of China to standardize injections made from TCM.

Many methods are currently being used in the study of fingerprinting including chromatography [14], capillary electrophoresis (CE) [15], high-performance liquid chromatography (HPLC) [16,17], and gas chromatography (GC) [18,19]. They have been widely considered the ideal methods in fingerprint analysis because of their many advantages. GC is outstanding for analyzing volatile components and HPLC is advantages for the analysis of the majority of chemical components of TCM. However, since chemical components of substances in TCM are so complex, it is very hard to use CE, HPLC, or GC to characterize all components. A rational approach to assure authenticity quality of products of TCM is essential.

High-speed countercurrent chromatography (HSCCC) is a form of liquid–liquid partition chromatography, which was first invented by Ito [20]. The liquid stationary phase is immobilized on the column using centrifugal force. When the mobile phase is pumped through the column, sample components are partitioned between the two phases, and they are separated on the basis of differences in partition coefficients. Compared with traditional liquid–solid column chromatography, use of HSCCC eliminate irreversible adsorption losses of samples and produce higher yields and efficiencies. Therefore, it is very suitable for the separation of active components from plants used in TCM and other natural products. Many successful applications of HSCCC have been reported. These include the separation of various components including alkaloids [21,22], flavonoids [23], polyphenols [24], terpenoids [25], antibiotics [26], and coumarins [27]. In this study, HSCCC was successfully used to fingerprint *P. corylifolia* L. The main chemical constituents of *P. corylifolia* L. were compared in different batches from different origins. The HSCCC fingerprint of *P. corylifolia* L. was established, and a comprehensive quality evaluation of similarity was calculated, which provides a reliable basis for controlling the quality of *P. corylifolia* L. in the future. Fingerprint analysis, as a method for the overall evaluation of plants used in TCM, can be used to identify the authenticity of plant products and evaluate the merits and demerits of *P. corylifolia* L.

## 2. Results

### 2.1. Selection of Extraction Methods

In the extraction process, extraction efficiency was affected by the use of different solvents. Since extracts of *P. corylifolia* L. are soluble and show obvious pharmacological effects in ethanol, ethanol is often used for the extraction and separation of extracts from the plant. Methods used for the extraction of the chemical components of *P. corylifolia* L. include cold soaking, water decoction, and ultrasonic extraction. Table 1 shows that the ultrasonic extraction was clearly superior to the other two methods used here. Only small quantities of psoralen and bavachin were extracted using the traditional decoction method. Further, levels of psoralen and bavachin produced using ethanol extraction and ultrasound were 5–7 times higher than those produced using water decoction. The stability of traditional decoction was affected by temperature, so the chemical structure and physicochemical properties of coumarin will be different. Therefore, the traditional decoction was not suitable for extracting chemical components of *P. corylifolia* L. As is shown in Table 1, ultrasonic extraction was superior with respect to the extraction of total compounds from *P. corylifolia* L. Therefore, components of *P. corylifolia* L. were extracted using ultrasonic extraction.

### 2.2. Optimization of Extraction Conditions

Results of the orthogonal experiment have been displayed in Table 2. According to max-min (R) values shown in Table 2, the order of factors that influenced the extraction efficiency of psoralen was A > C > B. Experimental results showed that ethanol concentration was the most important factor, and time of ultrasonic extraction was the second most important factor affecting psoralen extraction efficiencies. The order of factors affecting the extraction efficiency of bavachin was C > A > B. This data showed that the duration of the ultrasonic extraction was the most important factor affecting extraction efficiency. Further analysis of variance showed that ethanol concentration and the duration ultrasonic extraction greatly affected the extraction of both psoralen and bavachin (*p* < 0.01). According to the average factor level value (*K* value) shown in Table 2, A_3_B_1_C_3_ provided the best condition for performing ultrasonic extractions. The optimum extraction conditions were verified in repeated experiments. Therefore, A_3_B_1_C_3_ was selected as the condition used for the ultrasonic extraction of the chemical components of *P. corylifolia* L. samples. The extraction efficiency of psoralen and bavachin was greatest using this set of conditions compared to results of experiments using different orthogonal designs. When the optimal conditions were used, extracted concentrations of psoralen and bavachin were 4.55 and 3.94 ug/mL, respectively.

### 2.3. Selection of Two-Phase Solvent System

Most chemical components of *P. corylifolia* L. are moderately polar, therefore, solvent systems of hexane/ethyl acetate/methanol/water are often selected for HSCCC-based separation of the chemical components of the plant. In order to achieve the optimal resolution of target compounds, the polarity of each target should be considered. Five two-phase solvent systems were tested in which each had different ratios of *n*-hexane, ethyl acetate, methanol, and water. Ratios of *n*-hexane, ethyl acetate, methanol, and water (see Supplementary Materials) tested were as follows: 5:5:5:5, 5:5:6:4, 5:5:6.5:3.5, 4:6:6.5:3.5, 5:5.5:6.5:3.5, *v*/*v*/*v*/*v*). The *K*-values of compounds using each solvent system were determined using HPLC methods. Determination of *K*-values was summarized in Table 3.

The *K*-values of the six peaks within crude samples have been displayed in Table 3. When *n*-hexane–ethyl acetate–methanol–water (5:5:5:5), *n*-hexane–ethyl acetate–methanol–water (5:5:6:4), and *n*-hexane–ethyl acetate–methanol–water (4:6:6.5:3.5) were used as the solvent system, *K*-values of some peaks were more than 2, and good separation of some compounds was not achieved. When using a solvent system consisting of *n*-hexane–ethyl acetate–methanol–water (5:5:6.5:5), separation and the time required for acceptable separation of five compounds was achieved. However, the *K* value of compound 3 under these conditions was slightly elevated and, therefore, was not suitable for separation of the components of *P. corylifolia* L. *K*-values ranging between 0.5 and 2 indicated that each compound could be well separated using the *n*-hexane-ethyl acetate-methanol-water (5:5.5:6.5:5) solvent system. Therefore, the solvent system of *n*-hexane–ethyl acetate–methanol–water with the volume ratio of 5:5.5:6.5:5 was finally selected in order to isolate the target compounds using HSCCC.

### 2.4. Optimization of HSCCC Conditions

**Figure 2:**
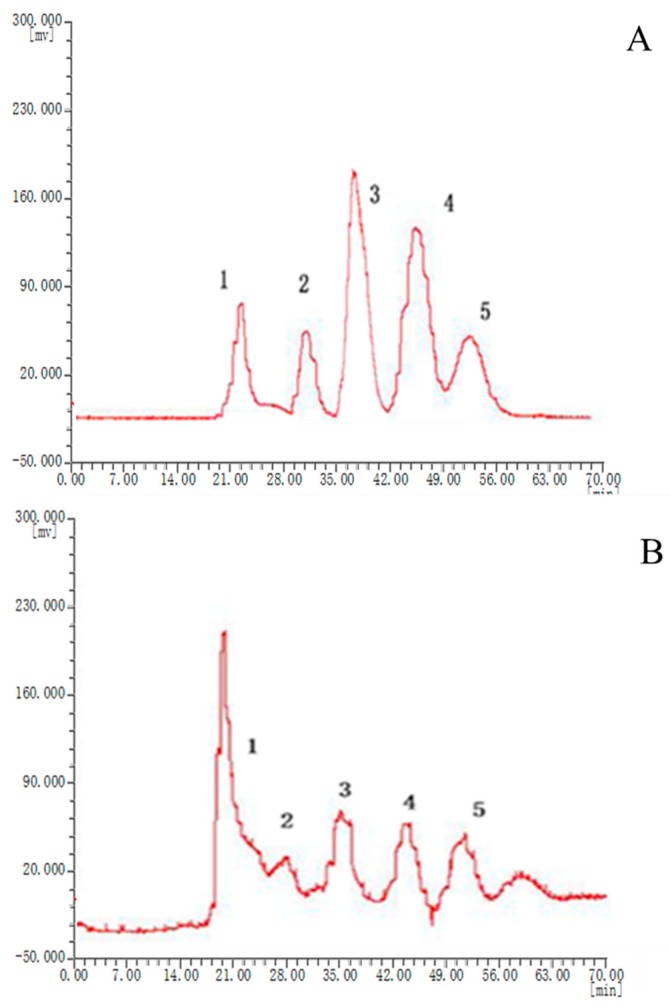
High-speed countercurrent chromatography (HSCCC) chromatogram of *Psoralea corylifolia* L. **A**: HSCCC chromatogram of standard compounds. **B**: HSCCC chromatogram of the crude sample of *Psoralea corylifolia* L. Peak 1: isobavachalcone; 2: bakuchiol; 3: isopsoralen; 4: psoralen; 5: bavachin. Conditions used to produce these results were as follows: Solvent system: *n*-hexane-ethyl acetate–methanol–water (5:5.5:6.5:5); speed: 1300 rpm; injection volume: 3.0 mg; flow rate: 1.0 mL/min.

Separation of compounds was also influenced by several HSCCC parameters including separation temperature, the flow rate of the mobile phase, revolution speed of the separation coil, and detection wavelength. Therefore, these parameters were also optimized in order to ensure adequate separation of the compounds. When the flow rate and revolution speed were set at 1.0 mL/min and 1300 rpm, the detection wavelength and sample concentration were set at 254 nm and 3.0 mg/mL, respectively, crude extract obtained baseline separation. Under these conditions, six fractions were produced, as shown in Figure 2 (see Supplementary Materials).

### 2.5. Methodological Verification

The reproducibility, precision, and stability of extractions using *P. corylifolia* L. extracts have been summarized in Table 4. The relative standard deviation (RSD) of the relative peak areas of each compound ranged between 1.21% and 3.00%. An investigation of the reproducibility of results revealed that the method produced a good level of reproducibility. To provide an evaluation of precision of the method, we measured the same sample solution six times and calculated its relative value. Results demonstrated that the RSD of the relative peak area was less than 2.62%, which indicated that this method was favorable with respect to precision. An analysis of stability (Table 4) showed that the RSD of relative peak area was less than 3.00%. This showed that samples were relatively stable for 24 h. Taken together, these data illustrated that the method was accurate, reproducible, reliable, and suitable for use in the fingerprint analysis of *P. corylifolia* L. (see Supplementary Materials).

### 2.6. Fingerprint of HSCCC

**Figure 3:**
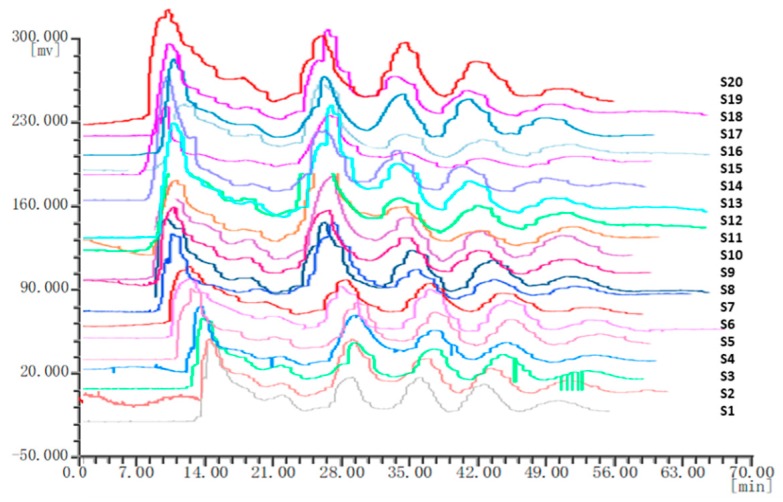
Twenty batches (S1–S20) of *Psorslea corylifolia* L. fingerprints.

**Figure 4:**
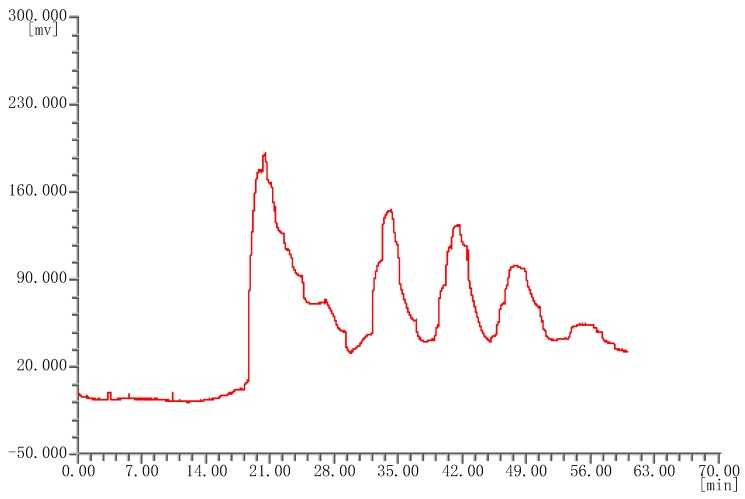
A curve that fits the fingerprint of *Psorslea corylifolia* L.

Twenty batches of *P. corylifolia* L. were analyzed by using “similarity evaluation system of chromatographic fingerprints of traditional Chinese medicine (2004A)” software. Fingerprints and similarity values (see Supplementary Materials) were generated after data processing. Six common peaks were shown in the fingerprint spectrogram from Figure 2. The chemical composition of five compounds was identified through comparisons with chemical standards. The chemical composition of peaks 1–5 was identified as isobavachalcone, bakuchiol, isopsoralen, psoralen, and bavachin, respectively. Although there were only six common peaks in the fingerprint of *P. corylifolia* L., it can also prove the uniqueness of medicinal materials. In the study of quality control, the TCM can be determined by 4–6 peaks. Since peak 1 had the largest peak area, the peak area of peak 1 in the 20 batches of samples was set to 1, and the other peak areas were expressed as relative values (Table 5). It was discovered that there were some differences in the areas of the six peaks, especially the areas of peaks 3 and 4. And the peak areas of the other four peaks were consistent. This indicated that the composition of *P. corylifolia* L. extracts is affected by region and batch. The fingerprints of 20 different batches of *P. corylifolia* L. have been shown in Figure 3, and the curve created to best-fit fingerprints has been shown in Figure 4. The similarity between fingerprints of *P. corylifolia* L. was calculated and the results have been shown in Table 6. Data (Table 6) clearly demonstrated that the fingerprints created from 20 batches of *P. corylifolia* L. were highly similar. Similarity values determined for the 20 batches varied between 0.725 and 0.954 and revealed that the chemical components of *P. corylifolia* L. in the 20 batches tested tended to be stable and have high level of similarity.

*P. corylifolia* L. has been widely used in the treatment of many diseases because of its therapeutic effects. In recent years, some counterfeit products have appeared on the market. *P. corylifolia* L. is often misused because its appearance and features such as color, shape, size, etc. are similar to *A. theophrasti* Medic. and *C. pallida* Ait. These plants are commonly sold as counterfeit of *P. corylifolia* L. *A. theophrasti* Medic. is derived from the dry seed of *Malvaceae* plant, which has detoxifying and fever-reducing properties. *C. pallida* Ait. is derived from the dried seed of the leguminous plant *C. pallida* Ait., which has anticancer effects but is harmful to the liver. *P. corylifolia* L. and its counterfeits cannot be distinguished on the basis of shape and physical properties.

**Figure 5:**
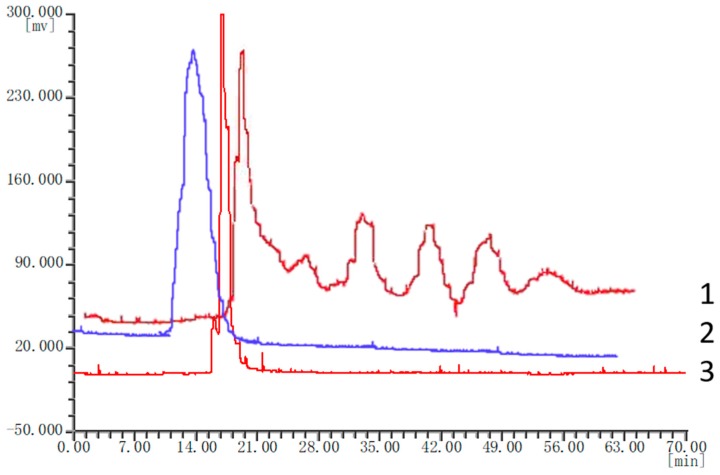
HSCCC chromatogram of *Psoralea corylifolia* L. (**1**), *Abutilon theophrasti* Medic (**2**), and *Crotalaria pallida* Ait. (**3**).

A HSCCC chromatogram (Figure 5) of three medicinal materials were compared. Figure 5 clearly shows that there are great differences in the chemical composition of the three medicinal materials. These results also illustrate that three types of therapeutics can be distinguished using the HSCCC method. Therefore, HSCCC method is a reliable way to identify *P. corylifolia* L. and its counterfeits easily and rapidly.

## 3. Discussion

The fingerprinting used in TCM provides a quality control index for the determination of individual components as well as the internal quality of the preparation as a whole. As a result, a comprehensive evaluation of the internal quality and comprehensive control of a wide range of TCM preparations is now possible. Fingerprinting provides a more comprehensive evaluation than individually measuring levels of an active component. In this study, the fingerprint of *P. corylifolia* L. was established using HSCCC. The fingerprint of *P. corylifolia* L. was analyzed using the software of the similarity evaluation system of fingerprint of traditional Chinese medicine (2004). Results showed that the quality of *P. corylifolia* L. can be affected by the region and batch from which a sample is derived. Although the same components were displayed in 20 batches of *P. corylifolia L.*, the growth environment and processing factors had certain influence on the levels of components extracted from *P. corylifolia* L. The average similarity between 20 batches derived from different locations was between 0.80 and 0.90 (Table 7). These results indicated that samples were highly similar and only small differences were observed within the 20 batches of *P. corylifolia* L. examined. Similarity among three batches from Henan and Zhejiang was greater than 0.90, which was higher than that of *P. corylifolia* L. from other areas. These results indicated that the quality of *P. corylifolia* L. in Henan and Zhejiang was better than that of other locations. Among the five batches collected from Sichuan, similarity varied greatly from 0.73 to 0.96 due to the different collection or production times, indicating that the quality of *P. corylifolia* L. from Sichuan was likely affected by the production process. Small differences within batches of *P. corylifolia* L. were observed in samples from Anhui, Henan, Hebei, and Yunan. Therefore, not only the authenticity but also the source and batches of *P. corylifolia* L. should be considered to ensure the proper identification and quality control of *P. corylifolia* L.

In this study, the HSCCC method was used in the fingerprint analysis of *P. corylifolia* L. The high resolution and reproducibility of HPLC and HSCCC are similar, so HSCCC is suitable for fingerprint of TCM, as it can be considered equivalent to HPLC. However, the cost of the HSCCC instrumentation and reagents required are lower than that of HPLC. Therefore, analytical HSCCC meets the needs of fingerprint studies and can be widely applied in the analysis of plants used in TCM. Today, there are many complex compounds used in TCM that stain chromatography columns. HSCCC is a support-free liquid–liquid partition chromatography method, which does not cause reversible adsorption. Moreover, samples were assessed without preliminary extraction before HSCCC. HSCCC is suitable for complex samples such as TCM and biological product preparations. The HSCCC fingerprint method was accurate, simple, and rapid and can provide a new technical method for fingerprinting. Use of the method has the potential to solve the problem of quality control for the internationalization of TCM. Taken together, our results indicate that HSCCC is an inexpensive, reliable, cost-effective, and feasible way to fingerprint products used in TCM and represents a new technology that may be used to control the quality of products used in TCM.

## 4. Experimental

### 4.1. Apparatus

HSCCC instrument employed in this study was an OptiChrome TM-30B analytical high-speed, countercurrent chromatograph (Counter Current Technology Co., Ltd., Beijing, China), which was equipped with two polytetrafluoroethylene analytical coiled separation columns connected in series (diameter of tube, 2.6 mm; total volume, 30 mL) with a 5 mL manual sample loop. The rotational speed of the apparatus was regulated with a speed controller that ranged between 0 and 1500 rpm. A Model UVD-680-4 detector (Jinda Biotech, Shanghai, China) and a model of V4.0 work station (Jinda Biotech, Shanghai, China) were used in the detection of the effluent at a wavelength of 254 nm.

The high-performance liquid chromatography (HPLC) (Shimadzu, Kyoto, Japan) equipment utilized a Shimadzu LC-10A system. Chromatographic analysis was carried out using a Kromasil C_18_ column (250 × 4.6 mm id 5 µm, Thermo Fisher Scientific, Waltham, MA, USA). Water (A) and methanol (B) (purity > 98%) were selected as mobile phases. Gradient elution was employed in the process. The conditions were as follows: 0–40 min, 40–100% B. The flow rate was 1.0 mL/min. The detector wavelength was set at 245 nm.

The fruits of *P. corylifolia* L. were ground into a fine powder by using a multifunctional crusher (Jinsui Machinery Manufacturer, Dalian, China, 28,000 rpm/min). Ultraviolet spectrophotometry employed a silicon light diode, a deuterium lamp, and a tungsten lamp (SP-752, Shanghai Spectroscopy Instrument Co., Ltd., Shanghai, China). The extraction of *P. corylifolia* L. was used by ultrasonic machine (Kudos, Shanghai, China).

### 4.2. Reagents and Materials

Solvents used for the extraction and preparation of crude samples, and for HSCCC-mediated separation of components, were of analytical grade (Sinopharm Chemical Reagents, Shanghai, China). Methanol used for the HPLC analysis was of chromatographic grade and was obtained from Sigma, USA. Distilled water used here was generated by a water purification system (RSJ Scientific Instruments Co., Xiamen China).

Twenty batches of *P. corylifolia* L. were collected from distinct locations, which included main producing area, such as Sichuan, Anhui (Table 8). Standards including psoralen, isopsoralen, bakuchiol, bavachin, and isobavachalcone (purity > 98%) were purchased from Nanjing Jingzhu biological science and Technology Co., Ltd. (Nanjing, China).

### 4.3. Pretreatment of P. corylifolia L.

There are two main types of compounds within *P. corylifolia* L.: coumarins and flavonoids, for example, psoralen and bavachin. As a result, psoralen and bavachin were selected as the main compounds to be measured within *P. corylifolia* L. The fruits of *P. corylifolia* L. were ground into fine powder and dried for 6 h at room temperature and were subsequently stored in a dryer. A 3.0 g portion of the powder was weighed precisely, and extractions were performed using three different approaches: (1) Materials were soaked for 24 h via the addition of 20 mL 95% ethanol at room temperature; (2) 20 mL distilled water was added and the decocting extraction performed for 30 min; (3) The extract was collected through the addition of 20 mL 95% ethanol and an ultrasonic extraction was performed for 30 min. After the extraction, three extracted solutions were filtered using filter paper and were quantitatively transferred to a 100 mL volumetric flask. The extracts were concentrated, dried at 50 °C, and dissolved using methanol. The content of psoralen and bavachin within *P. corylifolia* L. fruits obtained by different extraction methods were determined using ultraviolet spectrophotometry. Standards (psoralen, isopsorale, bakuchiol, bavachin, and isobavachalcone) were precisely weighed and 1.0 mg/mL solutions were made using methanol. Then, standard solutions were absorbed and diluted with methanol to obtain a dilution series.

### 4.4. Orthogonal Design Optimization of Ultrasonic Extraction Conditions

Based on the results of the three extraction methods outlined in Section 4.1, the optimal extraction method, ultrasound extraction, was selected for extraction of components within *P. corylifolia* L. Extraction efficiencies can be influenced by many factors. In order to optimize conditions used to extract components from *P. corylifolia* L., three factors affecting ultrasonic extraction rates of psoralen and bavachin were selected: ethanol concentration (A), ethanol volume (B), and ultrasonication time (C). Three levels of each factor were tested using orthogonal design. The experiments were performed based on the construction of an L_9_ (3^3^) orthogonal table. Table 9 depicts the factors that influenced the extraction of components at the experimental level.

*P. corylifolia* L. powder (3.0 g) was added in a 100 mL conical bottle and was ultrasonically extracted for different durations and with the addition of different concentrations and volumes of ethanol. The extracts from *P. corylifolia* L. were evaporated and concentrated under reduced pressure at 50 °C. Psoralen and bavachin content were measured using ultraviolet spectrophotometry. The experiments were repeated three times in parallel.

### 4.5. Preparation of Crude Samples from P. corylifolia L.

In a clean 100 mL conical bottle, *P. corylifolia* L. fruits were ground into fine powder, and 9.0 g of powder was transferred to 90 mL 95% methanol and an ultrasonification was performed for an hour. Subsequently, the extract was filtered and cooled to room temperature. Extracted solutions were obtained, concentrated, and dried at 50 °C. The extraction was repeated three times and all extracted products were combined. The extract was then dissolved using methanol, dried and stored at refrigerator (4 °C) for the subsequent separation using HSCCC. The same extraction method was used in the study of *Abutilon theophrasti* Medic. and *Crotalaria pallida* Ait.

### 4.6. Selection of the HSCCC Solvent System

The ideal separation of compounds for HSCCC depends on the proper selection of a two-phase solvent system [28]. A suitable solvent system for HSCCC will simultaneously provide an adequate stationary phase and mobile phase. In order to select a suitable two-phase solvent system, the golden rules of HSCCC [27] and characteristics of target compounds should be considered. The two-phase solvent system was selected according to the partition coefficients (*K*) of the compounds. In this paper, *K* values were determined by HPLC. The measurement of *K* values was determined using different ratios of *n*-hexane–ethyl acetate–methanol–water solvents, prepared and thoroughly equilibrated in a separatory funnel at room temperature. After all solvent systems were prepared, 3.0 mg of the extract was dissolved in 2 mL of the upper of solvent system, then an equal volume of the lower phase was added to the solution while shaking thoroughly. When partition equilibrium was reached, a total of 2 mL of each phase was transferred into another centrifuge tube and filtered through a 0.45 µm membrane filter. Each sample solution (20 μL) was analyzed by HPLC. The *K* value was calculated according to the following equation:

*K* = A_1_/A_2_

where A_1_ is the peak area of the upper phase and A_2_ is the peak area of the lower phase.

### 4.7. Preparation of the Two-Phase Solvent System and Sample Solutions

Because the compounds exhibited a medium level of polarity, *n*-hexane–ethyl acetate–methanol–water was selected as the two-phase solvent system to be used in further experiments. The *n*-hexane–ethyl acetate–methanol–water (5:5:5:5), *n*-hexane–ethyl acetate–methanol–water (5:5:6:4), *n*-hexane–ethyl acetate–methanol–water (5:5:6.5:3.5), *n*-hexane–ethyl acetate–methanol–water (4:6:6.5:3.5), and *n*-hexane–ethyl acetate–methanol–water (5:5.5:6.5:3.5) were used for the HSCCC analysis. Each solvent was added in a separatory funnel and was thoroughly equilibrated at room temperature. After they were vigorously shaken and settled overnight to avoid emulsification, upper and the lower phases were separated and degassed by ultrasonication for 30 min before using. The upper phase and lower phase were used as the stationary phase and the mobile phase separately. The sample solution and standard sample solution for separation using HSCCC were prepared by dissolving 3.0 mg of samples and standards in 1.0 mL of the mixture consisting of equal volumes of both upper phase and lower phases (1:1, *v*/*v*) of the solvent system.

### 4.8. HSCCC Separation Procedure

In each round of separation, the upper (stationary) phase was pumped into the coiled HSCCC column at maximum flow rate of 30 mL/min. When the column was completely filled, the HSCCC apparatus was run at 1300 rpm, while the lower (mobile) phase was pumped at a flow rate of 1 mL/min into the column. After hydrodynamic equilibrium was established, 1 mL of the sample solution containing 3.0 mg of each sample was injected into the coiled tube through the injection valve. The effluent from the outlet of the column was continuously monitored by a UV detector set to measure signal at 254 nm, and a chromatogram was recorded. The temperature was kept at 25 °C. Twenty batches of *P. corylifolia* L. and sample standards were analyzed using HSCCC.

### 4.9. Methodological Verification

#### 4.9.1. Precision

According to the chromatographic conditions outlined in Section 2.1, the crude extract of *P. corylifolia* L. from the same batch was extracted 6 times. The peak areas of six peaks were recorded, of which five can be confirmed by standard products, and the relative standard deviation (RSD) values were calculated.

#### 4.9.2. Reproducibility

Six samples from the same crude extracts of *P. corylifolia* L. were determined as outlined in Section 2.1. Then the RSD values from six peaks were calculated.

#### 4.9.3. Stability

The same batch of crude extract of *P. corylifolia* L. was obtained, and the peak areas of six peaks were recorded after 0, 2, 4, 6, 8, and 12 h under the above 2.1 terms. 

### 4.10. Establishment of the Fingerprint of P. corylifolia L. and Its Use for Comparing the Chemical Content of P. corylifolia Samples

The software, similarity evaluation system for chromatographic fingerprint of traditional Chinese medicine (2004A) was used to analyze HSCCC chromatograms from 20 batches of *P. corylifolia* L., which were issued by the National Pharmacopoeia Commission. Using these samples, a control fingerprint was generated. The relative peak area of each chemical component within *P. corylifolia* L. samples and the similarity of each batch of *P. corylifolia* L. were calculated using information within the fingerprint, and similarities and differences between samples of different origin and batches of *P. corylifolia* L. were analyzed.

## 5. Conclusions

As China has become increasingly wealthy, TCM has gained popularity in the international market. Fingerprinting is an accurate method used to evaluate the quality of traditional Chinese medicinal products. At present, the HSCCC analysis method can be widely used to perform quality control analyses of products used in TCM; since it is capable of separating components of mixtures at high levels of resolution, cost to perform procedures is low and no strict method required to pretreat of samples. In this paper, existing fingerprint methods were evaluated for use in the quality control of *P. corylifolia* L. extracts. As a result, a simple, low-cost, fast, and efficient fingerprint method was described using HSCCC was established to provide a basis to evaluate the quality products used in TCM. However, because of the variety and complexity of TCM, the HSCCC method can be further studied, and more coumarins and other Chinese medicines rich in other ingredients can be popularized to establish the fingerprint of TCM of HSCCC. The quality of TCM will be improved by using HSCCC. Moreover, the HSCCC technology can be optimized to better applied to the study of the fingerprint of TCM.

## Figures and Tables

**Table 1:** Effects of different extraction methods on the levels of psoralen and bavachin within total extracts of *Psorslea corylifolia* L. (*n* = 3).

	Content/(mg × g^−1^)		
Extraction Method	Psoralen	Bavachin	The Sum of Two Compounds
Ethanol leaching	13.27	8.75	22.02
Decoction extraction	3.35	0.84	4.19
Ethanol ultrasonic extraction	19.42	10.36	29.78

**Table 2:** L_9_ (3^3^) orthogonal test design and UV determination results (*n* = 3).

Test Number	A	B	C	(1) Psoralen ug/mL	(2) Bavachin ug/mL
1	1	1	1	1.15	1.07
2	1	2	2	1.53	1.27
3	1	3	3	0.81	2.01
4	2	1	1	0.87	2.21
5	2	2	2	1.96	3.49
6	2	3	3	4.07	2.97
7	3	1	1	2.96	3.57
8	3	2	2	2.86	1.65
9	3	3	3	2.67	0.98
K_1_	1.16	1.66	2.70	Psoralen	
K_2_	2.30	2.12	1.69		
K_3_	2.83	2.52	1.91		
R	1.67	0.86	1.00		
K_1_	1.45	2.28	1.85	Bavachin	
K_2_	2.89	2.14	1.49		
K_3_	2.07	1.99	3.02		
R	1.44	0.30	1.54		

**Table 3:** The partition coefficient (*K*) of target compounds determined using different solvent systems.

Solvent Systems (*v*/*v*)	*K* Value					
	1	2	3	4	5	6
*n*-hexane–ethyl acetate–methanol–water (5:5:5:5)	2.17	2.83	7.82	6.5	2.2	2.41
*n*-hexane–ethyl acetate–methanol–water (5:5:6:4)	1.17	1.58	2.73	2.43	1.10	2.29
*n*-hexane–ethyl acetate–methanol–water (4:6:6.5:3.5)	2.01	2.43	6.50	1.62	5.54	4.22
*n*-hexane–ethyl acetate–methanol–water (5:5:6.5:3.5)	1.26	1.46	3.01	1.70	1.28	1.57
*n*-hexane–ethyl acetate–methanol–water (5:5.5:6.5:5)	1.53	1.44	1.39	1.22	1.07	0.99

**Table 4:** The reproducibility, precision, and stability of extractions using *Psorslea corylifolia* L. extracts.

Compound	Precision RSD (%)	Reproducibility	Stability
		RSD (%)	RSD (%)
1	1.18	1.21	1.50
2	2.16	2.42	2.81
3	2.62	2.94	2.73
4	1.84	2.29	2.26
5	2.58	1.83	2.54
6	2.03	1.90	3.00

**Table 5:** Relative area of peaks determined using *Psorslea corylifolia* L. extracts.

Number of Peaks	1	2	3	4	5	6
S1	1	0.03	0.51	1.15	0.38	0.11
S2	1	0.04	1.14	0.78	0.55	0.11
S3	1	0.03	0.47	1.19	0.38	0.08
S4	1	0.03	0.56	1.31	0.40	0.10
S5	1	0.03	0.58	1.55	0.60	0.12
S6	1	0.06	0.53	1.46	0.50	0.10
S7	1	0.03	0.50	1.32	0.46	0.06
S8	1	0.04	1.35	0.66	0.49	0.19
S9	1	0.03	2.04	0.71	0.44	0.07
S10	1	0.02	0.96	0.37	0.23	0.06
S11	1	0.01	0.45	1.08	0.36	0.13
S12	1	0.13	0.64	0.60	0.40	0.16
S13	1	0.03	1.37	0.57	0.42	0.15
S14	1	0.03	1.69	0.43	0.13	0.13
S15	1	0.05	0.74	0.69	1.48	0.17
S16	1	0.02	1.47	0.41	0.45	0.21
S17	1	0.02	1.34	0.28	0.55	0.21
S18	1	0.04	1.61	0.38	0.09	0.20
S19	1	0.03	1.52	0.67	0.57	0.33
S20	1	0.05	0.52	1.14	0.47	0.21

**Table 6:** Similarity of *Psorslea corylifolia* L.

Number	Similarity
S1	0.90
S2	0.82
S3	0.92
S4	0.86
S5	0.91
S6	0.96
S7	0.95
S8	0.89
S9	0.76
S10	0.79
S11	0.83
S12	0.82
S13	0.73
S14	0.72
S15	0.75
S16	0.89
S17	0.82
S18	0.84
S19	0.82
S20	0.90

**Table 7:** Similarity of *Psoralea corylifolia* L. collected from different locations.

Place of Origins	Batch Number	Similarity	
		Range	Average
Sichuang	1, 6, 13, 14, 15	0.73–0.96	0.82
Anhui	2, 9, 18, 19	0.76–0.84	0.81
Henan	3, 5	0.91–0.92	0.92
Hebei	10, 11, 17	0.79–0.83	0.81
Yunnan	4, 12, 20	0.82–0.90	0.86
Guangxi	8, 16	–	0.89
Zhejiang	7	–	0.95

**Table 8:** Twenty batches of *Psoralea corylifolia* L. collected from distinct locations.

Number	Place of Origin	Batch Number
1	Sichuang	180410
2	Anhui	180513
3	Henan	170529
4	Yunnan	180618
5	Henan	161125
6	Sichuang	170901
7	Zhejiang	160316
8	Guangxi	180608
9	Anhui	170918
10	Hebei	170601
11	Hebei	170901
12	Yunnan	170101
13	Sichuang	161121
14	Sichuang	170824
15	Sichuang	180510
16	Guangxi	171107
17	Hebei	180617
18	Anhui	181020
19	Anhui	160710
20	Yunnan	170521

**Table 9:** Factors evaluated to optimize ultrasonic extraction conditions.

Factor Level	A/%	B/mL	C/min
1	50	20	20
2	75	30	40
3	95	40	60

## Supplementary Materials

The following are available online, HSCCC procedure, methodological validation, and fingerprint analysis are available as supporting data.
