# Supplementary material for: Quality Control of Psoralea corylifolia L. Based on High-Speed Countercurrent Chromatographic Fingerprinting
Source: Molecules. 2020 Jan 9;25(2):279. doi: 10.3390/molecules25020279 (PMC7024294; doi:10.3390/molecules25020279)
Supplement: Supplementary file 1 [file molecules-25-00279-s001.zip › molecules-679640-supplementary/fingerprint of Psoralea corylifolia L.docx]

批次1

批次2

批次3

批次4

批次5

批次6

批次7

批次8

批次9

批次10

批次11

批次12

批次13

批次14

批次15

批次16

批次17

批次18

批次19

批次20
