# Supplementary figures and images for: Quality Control of Psoralea corylifolia L. Based on High-Speed Countercurrent Chromatographic Fingerprinting
Source: Molecules. 2020 Jan 9;25(2):279. doi: 10.3390/molecules25020279 (PMC7024294; doi:10.3390/molecules25020279)

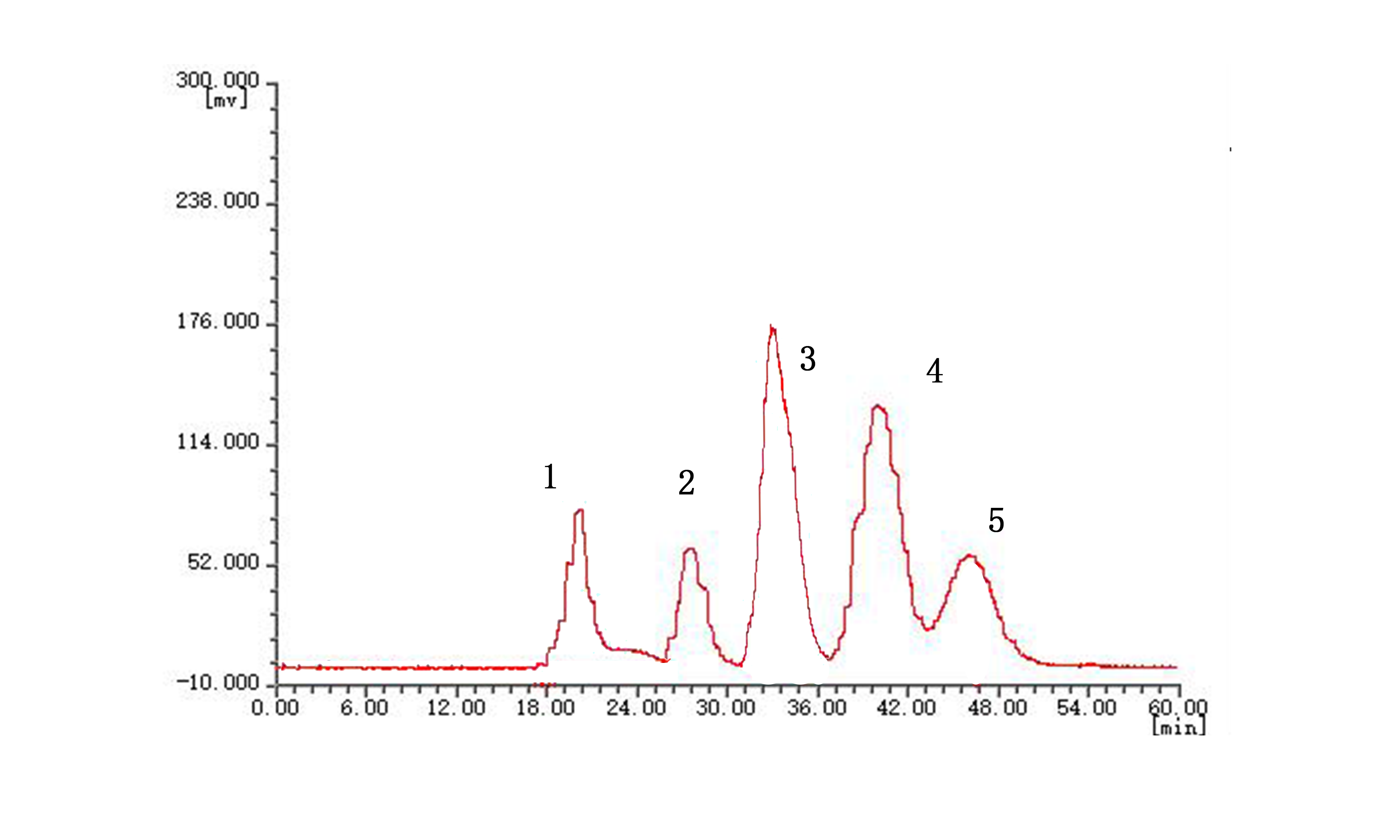

Supplement: Supplementary file 1 [file molecules-25-00279-s001.zip › molecules-679640-supplementary/Standard.png]
